# Sublime and extended reality experiences to enhance emotional wellbeing for autistic people: A state of the art review and narrative synthesis

**DOI:** 10.1177/00207640241261172

**Published:** 2024-07-24

**Authors:** Chris Bennewith, Johara Bellali, Lance Watkins, Samuel Tromans, Kamaldeep Bhui, Rohit Shankar

**Affiliations:** 1 Faculty of Arts, Humanities and Business University of Plymouth, UK; 2 University of South Wales, Pontypridd, UK; 3 Swansea Bay University Health Board, Port Talbot, UK; 4 Cornwall Intellectual Disability Equitable Research, University of Plymouth Peninsula School of Medicine, Truro, UK; 5 Department of Population Health Sciences, University of Leicester, UK; 6 Leicestershire Partnership NHS Trust, UK; 7 Department of Psychiatry, University of Oxford, UK; 8 Cornwall Intellectual Disability Equitable Research, Cornwall Partnership NHS Foundation Trust, UK

**Keywords:** Immersive experiences, neurodevelopmental disorders, pervasive developmental disorders, technology supported care, sublime

## Abstract

**Background:**

*Sublime* is a centuries old concept of emergent experience arising from immense and threatening awareness provoked by overwhelming fear and dread when faced with an incomprehensible situation as is common to autistic people. Extended Reality (XR) technologies have been used since the mid-1990s, in regulating emotions, behaviour and supporting social skill development for autistic people.

**Aims:**

To understand utility of XR technologies in creating immersive experiences for autistic people to alleviate anxiety and the relationship to the sublime.

**Method:**

A *State of the Art* literature review and narrative synthesis was conducted. PubMed, CINAHL, EMBASE, Cochrane Library, Scopus, Web of Science were searched with terms *Autism* AND *Technology*. In addition, fields of digital technologies and wellbeing, digital art and mental health, generative arts and the sublime were explored through web searches of grey literature, conversations with digital designers and explorations of extended reality platforms. No time limits were placed. Searches were done in English. Papers were screened and shortlisted using the inclusion criteria applied by two reviewers.

**Results:**

Fifty-eight papers/articles met the preliminary inclusion criteria for in-depth review of which 31 were found suitable for the narrative synthesis related to XR technologies and sublime experiences as related to autistic people. Narrative synthesis lent itself to four themes that is current utility of *XR Technologies* in autism, the impact of immersive experiences on *Behavioural, phenomenological and biological markers* of autistic people, the *Benefits of increased sensory stimulation* using XR on autism and an inquiry into the potential of *the sublime* for autism.

**Conclusions:**

Mixed reality environments that experiment with a broad range of XR technologies including incorporating notions of the sublime, might be beneficial in reducing emotional dysregulation and improving social development in autistic people especially if co-designed with them.

## Introduction

Approximately 0.6% of the global population are autistic (Salari et al., 2022). Autism prevalence is higher in certain groups, such as people with intellectual disability (Brugha et al., 2016), as well as adults using mental health services (Brugha et al., 2020). Autistic people are a highly heterogeneous population (Koehler & Falter-Wagner, 2023). Autistic girls have often been misdiagnosed or experienced delayed diagnosis as autistic features may manifest differently than in boys (Gesi et al., 2021). Characteristics in autistic people from diverse ethnic backgrounds or languages could also be misunderstood (Tromans et al., 2021). Autistic people are at a higher risk of mental and physical illness relative to their non-autistic peers (Croen et al., 2015; Lai et al., 2019).

### Autism’s triad of impairment

#### Social communication

Autistic individuals may struggle with language that is unclear, abstract, uses innuendos or sarcasm and they can be seen as having a rigid and literal understanding (Baxter et al., 2015; Kalandadze et al,2018). A study by Crompton et al. (2020) found that peer to peer communication between autistic individuals is highly effective, whereas lacking between neurotypical and neurodivergent individuals. Milton (2012) coined this as the double-empathy problem, which criticises the framing of autism as a communication disorder held uniquely by the autistic individual without investigating the interaction between autistic and non-autistic people, as a mutual, interpersonal issue. There is a gap in our understanding of the root causes of the misunderstandings and misperceptions that autistic people can experience, which can have an impact on their mental health and wellbeing (Milton, 2012). De Jaegher’s (2021) framework of *participatory sense-making*, an embodied relational inter-subjectivity that invites an attitude of ‘humility in the face of difference, is proposed as a way to build rapport and understanding and not assume a lack of capacity for understanding’(Milton et al., 2022). This opens a fascinating discussion in the arts, sociology and philosophy about how to develop shared experiences with autistic people and non-autistic people to develop empathic understanding of autistic experience (Milton et al., 2022).

#### Sensory abnormalities and restricted, repetitive behaviours, interests or activities

Kilroy et al. (2019) estimate that over 90% of autistic individuals show symptoms of sensory abnormalities. The general agreement (The Autism Group, 2018) is that three quarters of autistic individuals are suspected of sensory processing disorder/differences (SPD), meaning that their senses (touch, smell, taste, sight, auditory, vestibular, proprioception) might be overstimulated (hypersensitive) or under stimulated (hyposensitive).

Beyond Autism (2023) refers to sensory processing as ‘how the nervous system receives and interprets messages through the various senses and turns them into motor and behavioural responses’. An autistic individual may interpret sensory messages differently which may lead to challenges in managing behaviour and anxiety. They may need occupational therapists to support their regulation and sensory stimulation; they might have difficulty with motor skills, sense of balance and navigating around obstacles.

They might also show obsessive or repetitive behaviour, for example being focussed on one topic that absorbs all their interest or different forms of stimming, rocking or other repetitive behaviour that regulates their nervous system. Often, they thrive in routine or with a clear communication of what is to be expected, unpredictability can create distress.

#### Social interaction

One of the characteristics of autism that is often highlighted is a lack of emotional recognition in others and in oneself (alexithymia; Berthoz & Hill, 2005) and a deficient Theory of Mind (Baron-Cohen, 2000) – the ability to attribute mental states to ourselves and others. This hampers appropriate social interactions and social acceptance; autistic individuals can be judged as awkward, which increases the risk of rejection or being taken advantage of. However, according to Fitzpatrick et al.’s (2018) research on social synchrony with autistic and non-autistic youth, there is ‘no one theory [that] has been able to account for theory of mind, motor skills and emotional recognition’. In addition, their challenged vestibular sense can make them struggle with motor skills and balance. This impacts on social synchrony, relevant in supporting communication and social behaviour (McNaughton & Redcay, 2020).

### Sensory support for autistic people using extended reality technologies

Various proactive strategies such as creating structured environments, providing sensory and visual supports have shown positive results in alleviating distress for autistic people (Raulston & Hansen, 2022). One strategy is the use of extended reality (XR) technologies, primarily as teaching aids and skills development (Newbutt, Bradley, et al., 2020). XR is a catch-all term to refer to augmented reality (AR), virtual reality (VR) and mixed reality (MR). XR technologies are intended to combine or mirror the physical world with a ‘digital twin world’ which users can interact with (Vasarainen et al., 2021). The increasing diversity and affordability of these XR technologies opens new and different possibilities for the proactive treatment of autistic individuals. Appendix 1 provides further details on these technologies.

### The evolution of sublime experiences

The concept of the sublime has developed over many centuries, but truly gained traction during the Enlightenment. Philosophers like Edmund Burke and Immanuel Kant laid the groundwork for a deeper understanding of the sublime as an aesthetic and emotional experience. This was subsequently depicted by the romantic artists of the late 18th to early 19th Century, appearing in the works of artists like J.M.W Turner, Caspar David Friedrich and William Burke.

In *A Philosophical Enquiry into the Origins of Our Ideas of the Sublime and the Beautiful* (1757), Edmund Burke conceptualises the sublime as an emergent experience arising from immense and threatening awareness provoked by the vastness of nature. According to Burke, such experiences can bring feelings of pleasure, fear and importantly, the sublime. Burke elevated the sublime to a state of feeling that can be awakened by the physical forces of nature.

Kant in his *Critique of Judgment* (1790) contested that the sublime relates to the unconscious state of mind experienced when a human is faced with something that is difficult to understand or control. He conceived sublime as an aesthetic space where the human observer is overwhelmed by the massive scale of a pictorial object, triggering the perception of pleasure, awe and dread.

Recent research into the connection between sublimity and fear using behavioural and physiological measures (Hur et al., 2020) have brought into question the connection of fear and dread with a sublime experience and outline that these experiences are much more to do with beauty and scale.

### XR technology and sublime

XR technologies have created a new digital sublime experience which shifts the notion of the sublime from feelings of pleasure, awe and fear awakened solely by the physical forces of nature. They take account of the interconnected physical and virtual worlds as a fluid relation of visuality that re-establishes ‘nature’ as a physical/virtual field of vision that transcends the fixed and monumental boundaries of sublime ‘nature’ as conceptualised by Kant (Skowronska, 2018).

### Sublime, XR technologies and autistic people

Through its various definitions and interpretations, at its base, the sublime is a feeling rooted in humans’ relationships to the world, both physical and virtual and what lies beyond them, which help formulate a better understanding of those worlds and us (Crowther, 1984).

Strickland (1996) explored how assistive technologies, virtual, augmented and mixed realities, robots and other digital tools could be used to support autistic individuals as aids in managing difficulties associated with navigating a society principally designed for non-autistic people. The redefinition of the relationship between real and virtual spaces afforded through XR technologies, has enabled artists to explore new ways of creating sensory experiences, including new sublime experiences (Skowronska, 2018). It is this sensation that new MR experiences, could assist with reducing autistic people’s anxiety and potentially addressing the ‘double-empathy problem’.^1^

Currently, many autistic people lack suitable psychosocial support, compounding health inequalities, yet there is little research into their needs (Newbutt et al., 2023). Thus, there is a compelling need for evidence-based, accessible and engaging resources to support the mental health of young people to provide early proactive intervention strategies.

To date, VR has been the preferred technology for use in this type of research, however there are newer developments with MR experiences which allow audiences and users to have a collective immersive experience and not be reliant on Head Mounted Displays (HMDs),^2^ which have proven problematic for some autistic people, creating issues such as cybersickness and sensory problems (Bradley & Newbutt, 2018).

The aim of this study is to review the evidence for immersive experiences for autistic people as timely proactive strategies to improve their mental health.

## Research questions

The review looks to discover the following

RQ1: What are the latest developments of XR use with autistic groups?

RQ2: What is the notion of the sublime in autism?

## Methods

As this study is at the intersection of Arts, Science (clinical and technological) and Humanities, it aims to use transdisciplinary research principles. A State of Art literature review and narrative synthesis methodology was adopted to provide a time-based overview of the current state of knowledge and suggest directions for future research (Barry et al., 2022). State of the art reviews focus on recently published literature to assess current matters. A state-of-the-art review will often highlight new ideas or gaps in research with no official quality assessment (Baguss, 2020). In keeping with State-of Art review methodology the results are organised in relation to how the understanding of the examined elements has evolved over time.

PubMed, CINAHL, EMBASE, Cochrane Library, Scopus, Web of Science were searched with terms *Autism* AND *Technology*. For *Autism*, autism spectrum disorder, developmental disorder and pervasive developmental disorder were also used. Similarly, for *technology*, synonyms of computers, mechanisation and robotics were considered. Boolean operator (AND) was used. Citation searching of shortlisted papers was conducted. No MeSH terms, keywords, truncation, wildcards or exact phrases were utilised.

The fields of artificial intelligence software and hardware were explored through grey literature. Finally, the landscape of digital technologies and wellbeing, digital art and mental health, generative arts and the sublime was explored through a combination of web searches of grey literature, conversations with digital designers and explorations of extended reality platforms. Proceedings from the International Society for Autism Research (INSAR) conference and the ASSETS conference (the premier forum for presenting research on the design, evaluation, use and education related to computing for people with disabilities and older adults) were used to triangulate research trends. The search was conducted by the researchers (CB & JB) aided by information specialists. Searches were done in May 2023. Identified abstracts were screened further for inclusion criteria, methodological issues and other appropriate characteristics. The inclusion criteria required the paper/article discuss XR technology or sublime in autism. No time limits were placed. Searches were done only in English. Papers shortlisted had the inclusion criteria applied against the full text version independently by two reviewers. CB & JB conducted the data extraction. Disagreements regarding eligibility of studies was resolved by discussion and consensus within the project team. In keeping with a State of Art review principles no quality assessment of papers was undertaken.

## Results

Fifty-eight papers/articles met the preliminary inclusion criteria for in-depth review of which 31 were found suitable for the narrative synthesis related to XR technologies and sublime experiences as related to autistic people.

### XR technologies

The review looked very specifically at the use of XR, an umbrella term for augmented reality (AR), virtual reality (VR) and mixed reality (MR).

Research has shown that autistic individuals have a preference for visual stimulation from electronic screens and video games and show an affinity to computers and technology (Lorenzo et al, 2022; Schmidt et al., 2021; Tarantino et al., 2023). This is due mainly to their predilection for systematic and predictable environments, the fact that they can generally process visual information more easily and that technology offers a safe space for interaction without the consequences inherent in real-life environments.

Different combinations of XR hardware and software are already used to support autistic individuals depending on whether the objective is one of diagnosis, education or therapy (Newbutt et al., 2023). Tarantino et al.’s (2023) systematic review of immersive applications counted six empirical studies done on the use of HMDs to support autism and those were published between 2010 and 2017, only two of those used technologies that were not Virtual Reality. The development of cheaper, more light weight and useable HMDs is now expanding the application of different immersive technologies (Lorenzo et al., 2022, 2023; Mesa-Gresa et al., 2018; Newbutt, Bradley, et al., 2020; Tarantino et al., 2023).

The benefit of new lighter weight HMDs that allow AR or MR experiences, and indeed bespoke MR environments like those being created by artists and designers, over traditional VR experiences, is that they usually provide peripheral vision and the affordance of digital content being overlayed on real life experiences, rather than being solely immersing the user in a virtual world. This provides the additional benefit of these experiences being social and involving more than one person. This will be an important facet of creating experiences that allow collective meaning making between autistic people and their non-autistic peers if future research is to attempt to address the *Double Empathy* imperative and improved social interaction through social synchrony.

The benefits of newer AR and MR technologies need further research to fully understand the impact of HMD’s and immersive environments on autistic children (Lorenzo et al., 2023) however, research to date shows high acceptability, useability, enjoyment of these types of technology by the autistic population (Lorenzo et al., 2022; Newbutt, Bradley, et al., 2020; Schmidt et al., 2021; Shahmoradi & Rezayi, 2022; Tarantino et al., 2023; Yang et al., 2018).

### Behavioural, phenomenological and biological markers

One of the difficulties for autistic individuals is to ‘read the room’, to understand the non-verbal cues in a conversation, body language, innuendos, sarcasm or social norms (American Psychiatric Association, 2013). This often leads to confusion, anxiety and isolation. Koehler and Falter-Wagner (2023) identify a lack of phenomenological or biological diagnostic markers for autism diagnosis and present how digital technologies, including sensors, can support this gap. Sensors and markers utilised as part of an XR experience can help better understand and assess what happens to the autistic person during social interactions.

One objective of this review was to inquire whether biological and/or behavioural signals using sensors could inform the impact of repeated immersive experiences over a period of time. Lorenzo et al.’s (2016) research on the emotional response of autistic individuals using Immersive Virtual Environments (IVE) would justify this inquiry with his conclusion that autistic individuals ‘show a significant presence of more appropriate emotional behaviours in the immersive environments in comparison with the use of desktop VR applications’ and that these skills were transferred from the IVE to real life environments.

However digital phenotyping, defined as the ‘moment-by-moment quantification of the individual-level human phenotype in situ using data from personal digital devices’ (Torous et al., 2016) is intrusive and might not be adapted to autistic people. Motion Energy Analysis (MEA) has on the other hand shown positive results for non-verbal behaviour assessment that is objective and constraint free when coupled with machine learning (Georgescu et al., 2019).

According to Alcañiz et al. (2020), very few studies have recorded and analysed the process of behavioural change over time using biological signals in the autistic population. Yang et al. (2018) provided one such study with high functioning autistic youth over a period of 5 weeks. This is some of the first evidence into neuroplasticity in adults showing an increase in emotional recognition and social response after Virtual Reality-Social Cognition Training using neural & behavioural change. Earlier research by Cirelli et al. (2014) shows how interpersonal synchrony improves pro-social and socially cohesive behaviours. This points to the benefits of using behavioural and biomarkers to research the process that an autistic person goes through during social interactions to devise best strategies to improve their social inclusion that benefits their mental health.

### Benefits of increased sensory stimulation

Autistic individuals benefit from increased sensory stimulation. Jayne Ayres developed an approach, now referred to as Ayres Sensory Integration, to support the ability of individuals to respond appropriately to sensory stimuli (Lane et al., 2019). Increasing sensory stimulation is considered therapeutic in hyposensitive individuals and sensory integration can support autistic individuals to register, modulate and integrate sensory information. This is turn allows them to produce more adaptive behavioural and motor responses and helps them to engage and participate in social activities.

The most common method of providing controlled sensory stimulation for autistic people currently is through Multi-Sensory Environments (MSEs; also known as a sensory rooms). These are specialised spaces that contain equipment to modify the sensory environment for people who use them, across all modalities. They are used internationally (Bozic, 1997; Chan & Chien, 2017; Cuvo et al., 2001) and are widely used with autistic children as they relate to the differences in sensory processing experienced by autistic people, which comprise part of the core autism symptomatology.

A good example of research incorporating sensory rooms is the MEDIATE project (Carreras, n.d). This fascinating artwork explored what happens when an autistic child is allowed to explore responsive technologies in a safe setting and it encouraged creative exploration, while still maintaining a clear cause-and-effect relationship (Gumtau et al., 2005).

Developed in 2005 by The Centre of Responsive Environment at the University of Portsmouth, MEDIATE created a multi-sensory environment as an interface between autistic and non-autistic individuals. Through movement, sound and touch, autistic individuals could modulate the floor and the different technologies on the walls to generate feedback via an input output digital system. The autistic individual thus expresses their sensory preferences and regulates their nervous system. The project was evaluated as a success showing that autistic children using MEDIATE gained a sense of agency; empathy and interpretation and that it promoted creative activity (Gumtau et al., 2005).

Physical sensory stimulation and sensory rooms are recognised proactive strategies that support social behaviour and as a result, improved social inclusion and mental health benefits. Research on VR sensory rooms is also slowly emerging, Mills et al. (2023) at the University of Sydney, Australia recently published research evaluating VR sensory rooms with adults with disabilities with very positive results. No evidence was found of any mixed reality sensory rooms.

#### Autism and the sublime

This review found no papers, or current research, on the link between sublime experiences and pro-active strategies for supporting autistic people; and further, found no references of the use of MR technologies and the sublime as proactive strategies for supporting autistic people. There were however numerous connections found between autism and the sublime in the realms of art, personal experiences and more broadly, treatment for mental health in the Victorian era.

Artistic interventions have proven effective in addressing behavioural, social and developmental challenges in children diagnosed as autistic (Schweizer et al., 2019) . Engaging in creative arts enables autistic children to express themselves through diverse mediums, providing a secure environment for communication and skill development (Bharathi, Venugopal, et al, 2019; Schweizer et al., 2019). The utilisation of various art materials (including digital technologies) offers a broad spectrum of sensory experiences, including auditory and tactile sensations (Bharathi, Jayaramayya, et al., 2019; Schweizer et al., 2019), and can also shed light on the levels of depression and anxiety (Li et al., 2019).

The sublime has been a recurring theme in art and literature and probably the most well-known example of it being brought to life is by artist JMW Turner during what is known as the Romantic era. At the time, Turner was thoroughly studying the works of John Robert Cozens, himself a patient at Bethlem Royal Hospital,^3^ which was crucial for Turner’s understanding of the sublime (Tromans, 2008).

As previously noted, many autistic people have heightened sensory sensitivities. The sublime, with its emphasis on overwhelming and awe-inspiring sensory experiences, can sometimes resonate with how autistic individuals perceive the world. For some autistic people, sensory experiences, such as the beauty of nature or the grandeur of certain architectural spaces (awe, fear and wonder), can evoke intense emotional responses akin to the sublime.

Historically, the Victorians often used sublime experiences in an attempt to regulate the nervous systems of patients with mental health problems. Both the Victorian concept of a ‘Change of Air’ (Morris, 2018) and the institutions and treatments the Victorians used to support mental health in the late 19th and early 20th century provide a good example of the complex intersection at the time between spatial/geographical concerns, the concept of the sublime and societal attitudes towards mental health (Tromans, 2008).

## Discussion

The literature review undertaken identified a substantial body of research which showed a high acceptance of XR technologies and their positive outcome in terms of cognitive indexes, social behaviour changes and the potential for emotional and nervous system regulation for autistic people. It highlighted that the use of these technologies since the late 90s has been widely used, however that their use has predominantly been oriented around VR technologies and the use of HMDs as opposed to exploring some of the newer MR technologies available whether commercial or proprietary.

Widening the use of these technologies opens up the opportunity to create more social immersive virtual experiences which don’t shut users off from each-other provides a platform for further research into Milton’s double-empathy imperative. Creating these types of MR experiences could be beneficial for both autistic and non-autistic people, sharing spaces for co-creation, co-exploration and collective meaning making, as long as there is a true recognition and acceptance of the different strengths and abilities, and the unique perspective autistic people offer.

The review highlighted that in the creation of these MR experiences that interacting with autistic individuals requires a nonjudgmental, compassionate, patient and curious attitude. As each autistic individual’s characteristics and severity are unique, it is essential to adopt a person-centred approach and be informed by their unique sensory profile and care plan. Autistic people experience the world differently to neurotypical people through their senses and their perspective or perception of their environment.

The review also uncovered a history of association of mental health and wellbeing with notions of the sublime and a new conception of the sublime which includes new digital technologies. Although there were no specific references of sublime experiences being used in pro-active strategies involving autistic people, it appears that it could be beneficial to research the effect of these experiences on the health and wellbeing of autistic individuals, as well as the potential to use them to address the issue of double empathy.

### Limitations

This review does not utilise structured reporting measures such as PRISMA or registration in PROSPERO. This is due to the interdisciplinary nature of the theme. However, to ensure a suitable proven methodology a state-of-the-art review structure has been used. As state-of-the-art reviews generally focus on highlighting new ideas or gaps in research no official quality assessment of the papers were made. Thus, the individual paper quality has not been the focus of this study which could influence some of the assertions made.

It is important to remember that the relationship between the sublime and autism is subjective and varies from person to person. Not all autistic people will have a connection to the sublime, and those who do may experience it in diverse ways. Exploring this connection can provide insights into the richness and diversity of human experiences and emotions, shedding light on the intricate ways people engage with the world around them.

A key point uncovered in the review, was that whilst MR technologies have been used for the past 30 years with autistic individuals, there has been a lack of inclusion of these individuals in the development and application of these new technologies. Co-creation and the inclusion of autistic people in any future research is deemed essential for it to be relevant to the needs of the autistic community.

## Conclusion

Whilst the utilisation of XR technologies has shown positive results for improving the lives of autistic people, empirical results still face significant challenges. References to the sublime and its relation to mental health issues were prevalent in the literature, however there was no reference found to the specific use of sublime experiences to support proactive strategies with autistic people.

Results outlined the opportunity for further interdisciplinary research to take place at the intersection of pro-active sensory strategies for supporting autistic people, XR technologies and sublime experiences, to fill a knowledge gap.

## Appendix 1

Augmented Reality (AR) overlays digital information and objects onto the real world, typically through a device like a smartphone or AR glasses, allowing users to interact with their physical surroundings while incorporating digital elements. Virtual Reality (VR), on the other hand, immerses users completely in a computer-generated environment, often using VR headsets, creating a sense of presence in a virtual world that is entirely separate from the real world. Mixed Reality (MR), also known as hybrid reality, merges elements of both AR and VR by integrating digital content into the real world and enabling interactions between the two (Flavián et al., 2019).

MR devices, like the Microsoft HoloLens, enable digital objects to interact with the physical environment and appear to coexist with it, enhancing the user’s perception and interaction with both realms. In essence, AR enhances the real world with digital overlays, VR creates a wholly virtual environment, and MR blends digital and physical elements, creating a unique and interactive experience that combines the best of both worlds.

Examples of these technologies include commercial offerings such as the Oculus Rift (https://en.wikipedia.org/wiki/Oculus_Rift), Magic Leap (https://www.magicleap.com/en-us/) and the HTC Vive (https://en.wikipedia.org/wiki/HTC_Vive), alongside more bespoke technologies being developed by artists and designers, amongst others.

## Notes

1. Milton (2012) outlines the `double empathy problem’ as an issue of autistic and non-autistic people not being able to perceive or understand each other, hence creating a failure of empathy in both directions. He suggests that part of the difficulty for autistic people fitting into society is not just that they misunderstand others, but that they are in turn are misunderstood.

2. In the context of this review, Head Mounted Displays relate to the generic term for headsets and range from simple technologies providing a Virtual Reality (VR), Augmented Reality (AR) or Mixed Reality (MR) experience.

3. Founded in 1247, Bethlem Royal Hospital is a psychiatric hospital in Bromley, London. The hospital is closely associated with King’s College London and, in partnership with the Institute of Psychiatry, Psychology and Neuroscience, is a major centre for psychiatric research.
